# Supplementary material for: Novel Driver Strength Index highlights important cancer genes in TCGA PanCanAtlas patients
Source: PeerJ. 2022 Aug 11;10:e13860. doi: 10.7717/peerj.13860 (PMC9375969; doi:10.7717/peerj.13860)
Supplement: Supplemental Information 2 [file peerj-10-13860-s002.zip › COHORTS/genes plots/cohorts/2021_8_16_14_9_distribution_cohorts_females_LUAD.pdf]

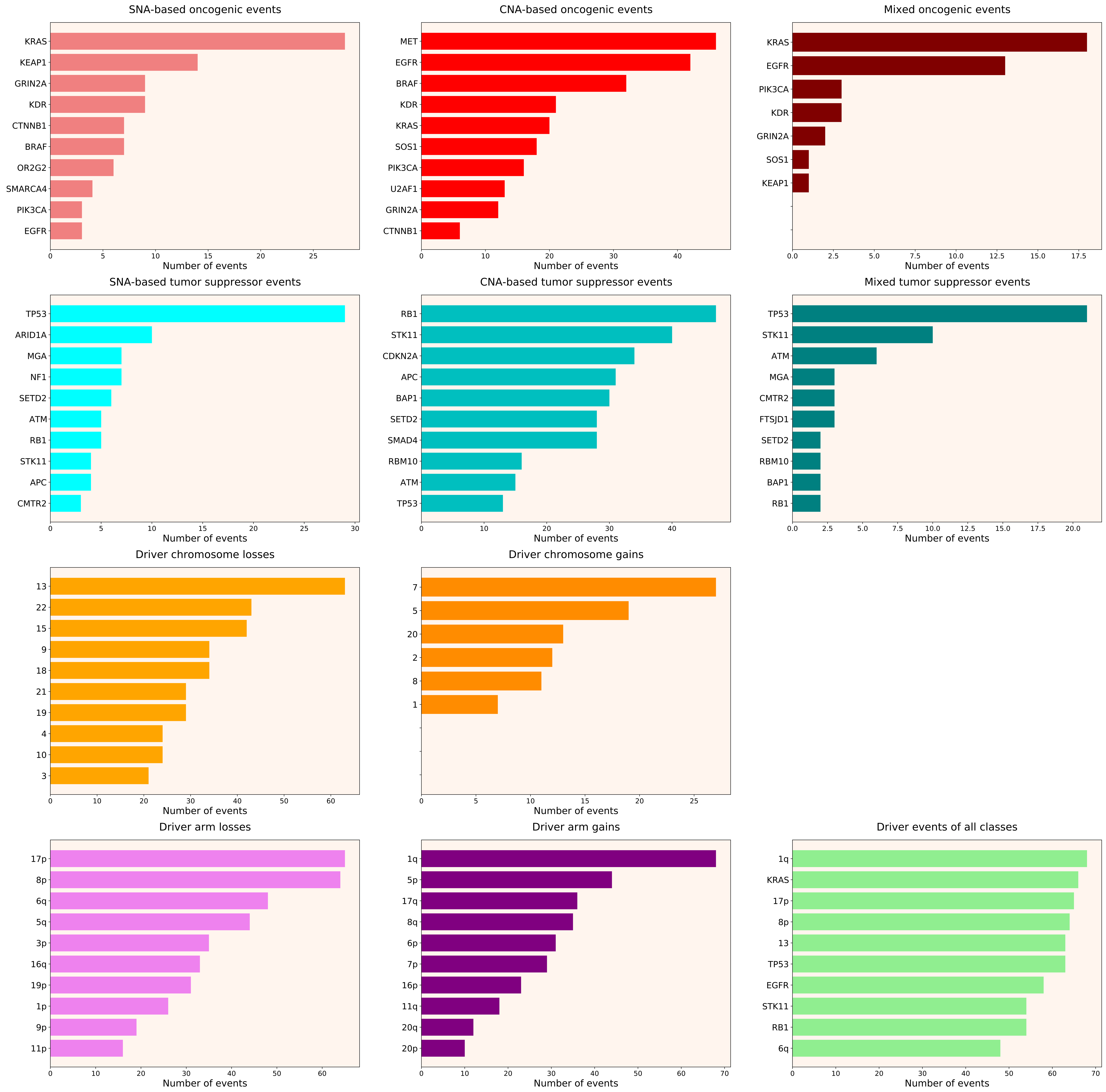
SNA-based oncogenic events
KRAS
KEAP1
GRIN2A
KDR
CTNNB1
BRAF
OR2G2
SMARCA4
PIK3CA
EGFR
0 5 10 15 20 25
Number of events
CNA-based oncogenic events
MET
EGFR
BRAF
KDR
KRAS
SOS1
PIK3CA
U2AF1
GRIN2A
CTNNB1
0 10 20 30 40
Number of events
Mixed oncogenic events
KRAS
EGFR
PIK3CA
KDR
GRIN2A
SOS1
KEAP1
0.0 2.5 5.0 7.5 10.0 12.5 15.0 17.5
Number of events
SNA-based tumor suppressor events
TP53
ARID1A
MGA
NF1
SETD2
ATM
RB1
STK11
APC
CMTR2
0 5 10 15 20 25 30
Number of events
CNA-based tumor suppressor events
RB1
STK11
CDKN2A
APC
BAP1
SETD2
SMAD4
RBM10
ATM
TP53
0 10 20 30 40
Number of events
Mixed tumor suppressor events
TP53
STK11
ATM
MGA
CMTR2
FTSJD1
SETD2
RBM10
BAP1
RB1
0.0 2.5 5.0 7.5 10.0 12.5 15.0 17.5 20.0
Number of events
Driver chromosome losses
13
22
15
9
18
21
19
4
10
3
0 10 20 30 40 50 60
Number of events
Driver chromosome gains
7
5
20
2
8
1
0 5 10 15 20 25
Number of events
Driver arm losses
17p
8p
6q
5q
3p
16q
19p
1p
9p
11p
0 10 20 30 40 50 60
Number of events
Driver arm gains
1q
5p
17q
8q
6p
7p
16p
11q
20q
20p
0 10 20 30 40 50 60 70
Number of events
Driver events of all classes
1q
KRAS
17p
8p
13
TP53
EGFR
STK11
RB1
6q
0 10 20 30 40 50 60 70
Number of events
